# Oxytocin Receptor Antagonists, Atosiban and Nolasiban, Inhibit Prostaglandin F_2α_-induced Contractions and Inflammatory Responses in Human Myometrium

**DOI:** 10.1038/s41598-019-42181-2

**Published:** 2019-04-08

**Authors:** Sung Hye Kim, Lucia Riaposova, Hauwa Ahmed, Oliver Pohl, André Chollet, Jean-Pierre Gotteland, Aylin Hanyaloglu, Phillip R. Bennett, Vasso Terzidou

**Affiliations:** 1 Imperial College London, Parturition Research Group, IRDB, Du Cane Road, London, W12 0NN UK; 2 ObsEva SA, R&D, Chemin des Aulx 12, CH-1228 Plan-les-Ouates, Geneva, Switzerland; 3 Present Address: André Chollet Consulting, CH-1295 Tannay, Switzerland

## Abstract

Oxytocin receptor antagonists (OTR-A) have been developed as tocolytics for the management of preterm labour due to the significant role of oxytocin (OT) in the onset of both term and preterm labour. Similar to OT, prostaglandins (PGs) play key roles in myometrial contractility and cervical ripening. Inhibition of PG synthesis/activity is used to delay preterm birth. Thus, targeting the PG pathway in combination with an OTR-A may be an effective strategy for delaying preterm delivery. In this study, we examined the effects of atosiban and nolasiban on PGF_2α_-induced contractions and pro-inflammatory responses in human pregnant myometrium. Both OTR-As, atosiban and nolasiban, inhibited PGF_2α_-induced contractions in a dose-dependent manner (*p* < 0.001 and *p* < 0.01, res*p*ectively). These inhibitory effects involved the suppression of PGF_2α_-mediated increase in intracellular calcium levels. In addition, the OTR-As significantly suppressed PGF_2α_-induced activation of pro-inflammatory pathways such as NF-κB and mitogen activated protein kinases (MAPKs), and the subsequent expression of contraction-associated-protein, COX-2. We have demonstrated that atosiban and nolasiban not only inhibit contractions elicited by OT, but also inhibit contractions and inflammation induced by PGF_2α_. This suggests a possible crosstalk between OTR and PG receptor signalling and highlights the importance of understanding G protein-coupled receptor interactions/crosstalk in the development of future tocolytics.

## Introduction

Preterm birth, defined as delivery before 37 weeks of gestation, is the major cause of perinatal morbidity and mortality worldwide^1^. Despite the growing focus in research, there is still limited understanding of the physiology of normal labour and preterm labour. Although a number of pharmacological agents also known as tocolytics have been introduced for the management of uterine contractions to delay preterm labour, there are no satisfactory tocolytics developed to date^2^ as there is no clear evidence that they improve neonatal outcome. However, tocolysis is considered for a few days to allow completion of a course of corticosteroids for fetal lung maturation and to allow *in utero* transfer^3^. Most tocolytics currently available for use are aimed to suppress uterine contractions, however, are not utero-specific and therefore can exert multi-organ fetal, neonatal and maternal side effects^4^.

Primarily, myometrial contractility is believed to be regulated by the changes in the levels of intracellular calcium^5–7^. An increase results in the activation of calmodulin and myosin light chain kinase (MLCK), which in turn triggers actin filaments and the generation of a contraction force. The reduction in the intracellular calcium levels is thought to decrease calmodulin and MLCK interaction leading to relaxation, however, the mechanism through which such transient increase in intracellular calcium levels and phosphorylation of MLC maintain the tonic force is yet to be understood^8^.

Oxytocin (OT) is a potent stimulator of myometrial contractions and plays an important role in the initiation of both term and preterm labour^9^. OT stimulates myometrial contractions through multiple signalling pathways^10^. Binding of OT to its receptor has been known to lead to G-protein coupling and subsequently, increase in intracellular calcium levels to mediate the generation of force^10^. Therefore, oxytocin receptor (OTR) is commonly used as a target for the development of tocolytics, and the only drugs developed specifically for the management of preterm labour are the OTR antagonists (OTR-As). Due to their increased specificity to the uterus, OTR-As such as atosiban can act as a suppressant of contractions with improved safety profiles^11^. Atosiban is primarily an arginine vasopressin (AVP) V1a receptor antagonist with lower affinity for the OTR. Its mechanism of action is via dose-dependent inhibition of OT-mediated increase in intracellular calcium levels which involves closing of voltage gated channels to prevent influx of calcium^12^. Atosiban has been approved in Europe for treatment of preterm labour but is administered through a bolus injection followed by an infusion and is not indicated for dosing beyond 48 hours^11^. Despite the previous reports of atosiban efficacy, it has been suggested to have a biased agonist effect in human amnion where it appears to act as a G_αq_ antagonist whilst activating G_αi_ signalling, leading to a pro-inflammatory response^13^. Nolasiban is a potent, selective, orally administered, non-peptide OTR-A with low affinity towards the AVP V1a and V2 receptors. Unlike atosiban, it was found to inhibit both G_αq_ and G_αi_ signalling induced by OT^14^.

Prostaglandins (PG) also play a central role in the onset as well as the maintenance of labour^15^. PGF_2α_ is a naturally occurring prostaglandin that acts to induce uterine contractions and labour in pregnant women. Currently available PG inhibitors such as the NSAID, indomethacin, act by non-selective inhibition of PG-forming enzymes, thus blocking the generation and signalling of many PG sub-types, including PGF_2α_. Because they potentially adversely affect fetal physiology, NSAIDs are no longer recommended for pregnant women^16^. Through PGF_2α_ receptors (FP receptors), PGF_2α_ can elevate intracellular calcium in myometrium^17^ and in turn, drive uterotonic effects that resemble an inflammatory response, whilst promoting the biophysical changes leading to cervical ripening^18^. Cervical ripening represents an integral part of the induction of labour process and prostaglandins such as dinoprostone are routinely used in clinical practice to promote cervical remodelling reducing the duration of labour and risk of caesarean section. In addition to promoting contractions and cervical ripening, it has been established that PGF_2α_ is able to trigger upregulation of uterine activation proteins such as COX-2, OTR and connexion-43 (CX-43)^19^. Antagonism of the FP receptor decreases uterine contractions, and prevents membrane ruptures and cervical changes, which are the key features of preterm labour resulting in preterm birth. FP antagonists, such as the peptide THG113.31, have been developed for the management of preterm labour^20^, however, their clinical efficacy is yet to be demonstrated.

Initially, studies in animal models have demonstrated that OTR antagonism can affect PGF_2α_-mediated signalling and vice versa^21,22^. The aim of this study was to investigate and compare the inhibitory effects of nolasiban and atosiban on spontaneous, OT-, and PGF_2α_-stimulated contractions in an *ex vivo* model using strips of pregnant human myometrium. Furthermore, we aimed to identify the secondary intracellular effects, such as changes in intracellular calcium levels and to assess whether nolasiban and/or atosiban can inhibit PGF_2α_-induced downstream pro-inflammatory responses in human myometrial smooth muscle cells.

## Results

### Inhibition of OT-induced myometrial contractions

**Figure 1:**
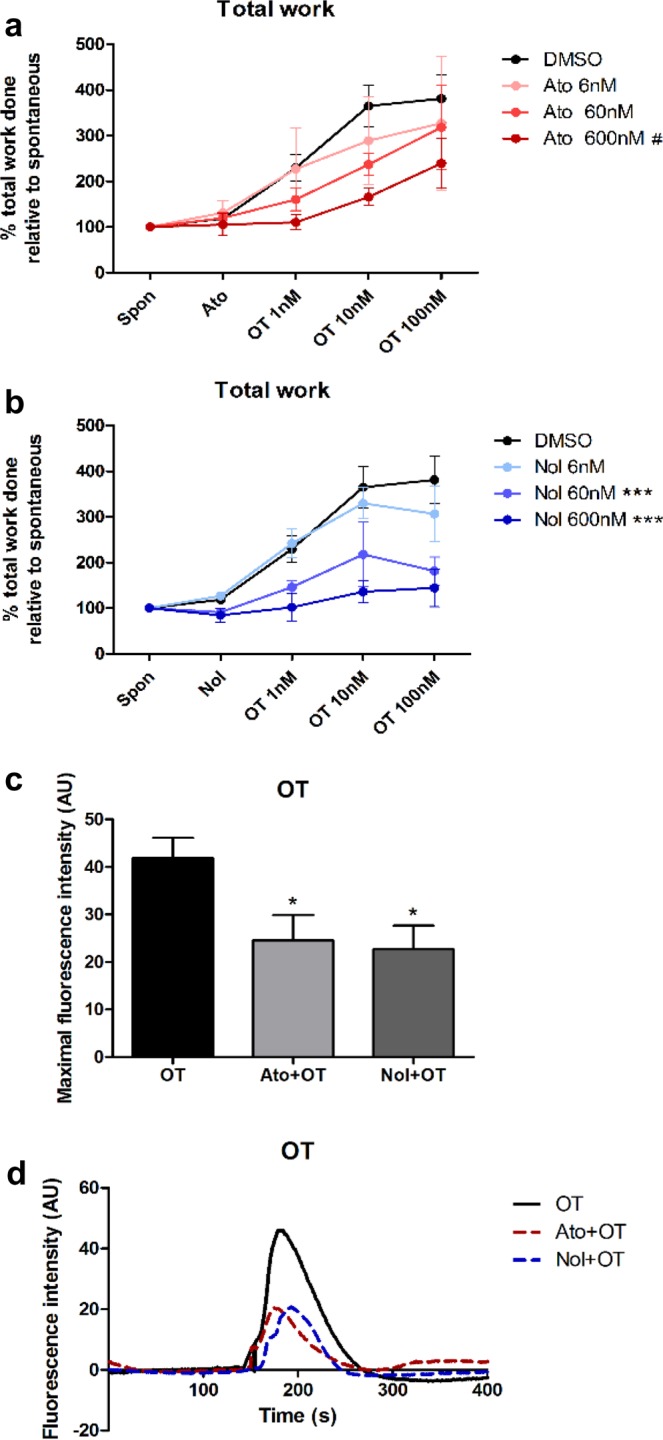
The effect of atosiban and nolasiban on spontaneous and OT-induced myometrial contractions. Pre-labour lower segment myometrial biopsies were subjected to stretch force of 4 g to attain spontaneous contractions. After 20 min of basal reading, vehicle control (DMSO), atosiban (Ato) or nolasiban (Nol) (6, 60, or 600 nM) was added and its effect on spontaneous contractions was measured for 10 min. The effect of the atosiban (**a**) or nolasiban (**b**) upon OT was then measured by adding increasing concentrations of the agonist (1, 10, and 100 nM) at 10 minute intervals. Total work (area under all contractions) was measured for each experimental time point and re-expressed as a ratio to the baseline period measurements (n = 6, ***p < 0.001 Nol vs DMSO; ^#^*p* < 0.05 Ato vs DMSO; ANOVA). Primary myometrial cells were loaded with calcium sensitive dye, Fluo-4-AM, prior to stimulation with OT (100 nM) in presence or absence of atosiban (600 nM) or nolasiban (600 nM) pretreatment. Maximal fluorescence intensities are expressed as mean ± SEM in arbitrary units (AU) (n = 6, *p < 0.05 vs OT; ANOVA) (**c**) and the fluorescence intensity profiles of a representative cell is shown in (**d**).

We observed a robust progressive increase in myometrial contractility with increasing doses of OT in all experiments (Fig. 1). Incubation with atosiban or nolasiban had no significant effect on spontaneous contractility when compared to its vehicle control, however, the stimulatory effect of OT was suppressed by atosiban (Fig. 1a) and nolasiban (Fig. 1b). Both produced a concentration-dependent inhibition of OT-induced myometrial contractility, significantly reducing the rate of contraction, and the average area under the curve (Supplementary Fig. S1). Nolasiban at 60 nM was sufficient to significantly decrease OT-induced myometrial contractility (*p* < 0.001 vs DMSO), whereas atosiban exerted its effects at 600 nM (*p* < 0.05 vs DMSO). When comparing equimolar concentrations, nolasiban appeared more potent than atosiban at 60 nM and 600 nM but this did not reach statistical significance.

The effects of OTR-As on OT-mediated calcium responses were examined by loading the myometrial cells with Fluo-4-AM calcium dye. We have confirmed that the calcium response driven by OT stimulation is consistent in myometrial cells throughout passages 0 to 10 (data not shown). Human myometrial smooth muscle cells at passages 4–7 were used to observe the changes in their agonist-mediated calcium response in presence or absence of the OTR-As. The results showed a consistent response to OT stimulation with a substantial increase in Fluo-4-AM fluorescence and hence, intracellular calcium concentration. The maximal fluorescence intensity induced by OT was suppressed in presence of both atosiban and nolasiban by 41.5% (*p* < 0.05) and 45.8% (*p* < 0.05), respectively (Fig. 1c). Figure 1d shows the representative fluorescence intensity profile of an individual cell in response to OT with/without pre-treatment with OTR-As.

### Inhibition of PGF_2α_-induced myometrial contractions

**Figure 2:**
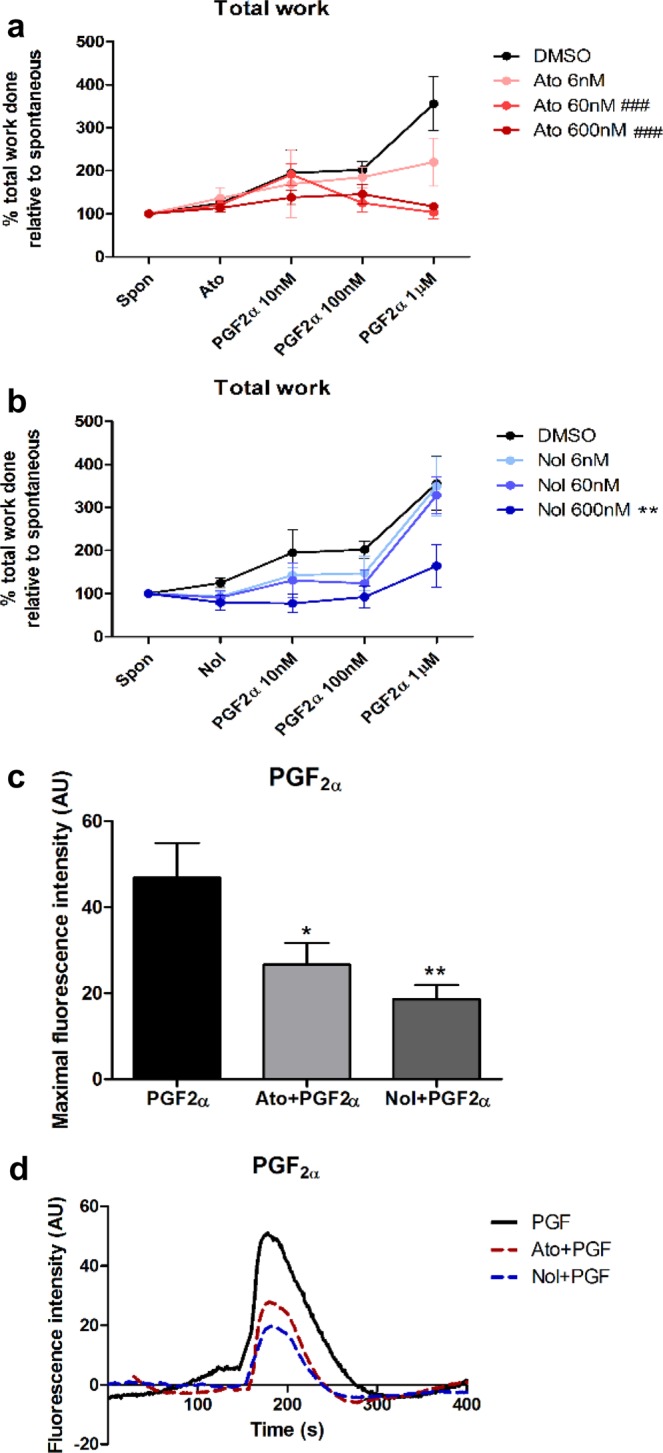
The effect of atosiban and nolasiban on PGF_2α_-induced myometrial contractions. Pre-labour lower segment myometrial biopsies were subjected to stretch force of 4 g to attain spontaneous contractions. After 20 min of basal reading, vehicle control (DMSO), atosiban (Ato) or nolasiban (Nol) (6, 60, or 600 nM) was added and its effect on spontaneous contractions was measured for 10 min. The effect of the atosiban (**a**) or nolasiban (**b**) upon PGF_2α_ was then measured by adding increasing concentrations of agonist (10, 100, and 1000 nM) at 10 minute intervals. Total work (area under all contractions) was measured for each experimental time point and re-expressed as a ratio to the baseline period measurements (n = 6, **p < 0.01 Nol vs DMSO; ^###^*p* < 0.001 Ato vs DMSO; ANOVA). Primary myometrial cells were loaded with calcium sensitive dye, Fluo-4-AM, prior to stimulation with PGF_2α_ (1 µM) in presence or absence of atosiban (600 nM) or nolasiban (600 nM) pretreatment. Maximal fluorescence intensities are expressed as mean ± SEM in arbitrary units (AU) (n = 6, *p < 0.05, **p < 0.01 vs PGF2α) (**c**) and the fluorescence intensity profiles of a representative cell is shown in (**d**).

There was a consistent dose-dependent enhancement of myometrial contractile performance with PGF_2α_ stimulation albeit to a lesser extent than OT (Fig. 2). There was a steady, dose-dependent increase in the overall contractile output (*p* < 0.001 at 1 µM vs spontaneous contractions), affecting the rate of contractions (*p* < 0.01 at 1 µM vs spontaneous contractions), average area under the curve (*p* < 0.05 at 1 µM vs spontaneous contractions), and peak amplitude (*p* < 0.01 at 1 µM vs spontaneous contractions) (Supplementary Fig. S2).

For both atosiban and nolasiban, we observed a significant reduction in PGF_2α_-induced rate of uterine contractions at 600 nM (*p* < 0.01 and *p* < 0.05, respectively). Nolasiban decreased the contraction duration, however, this did not reach significance. Atosiban (60 nM) reduced contraction peak amplitude by 43.3% (*p* < 0.01 vs DMSO) (Supplementary Fig. S2). The effect of PGF_2α_ on the total work done was inhibited by 60 nM atosiban and 600 nM nolasiban by 67.1% and 53.8%, respectively, compared to vehicle (DMSO) control (Fig. 2a,b).

In human myometrial cells, PGF_2α_ driven calcium response was reproducibly obtained. This agonist-induced changes in intracellular calcium levels were significantly suppressed by nolasiban (60.2% decrease, *p* < 0.01), and to a lesser degree, by atosiban (43.2% decrease, *p* < 0.05) (Fig. 2c). Figure 2d shows the representative fluorescence intensity profile of an individual cell in response to PGF_2α_ with/without pre-treatment with OTR-As.

### Inhibition of PGF_2α_-induced pro-inflammatory responses in human myometrial cells

**Figure 3:**
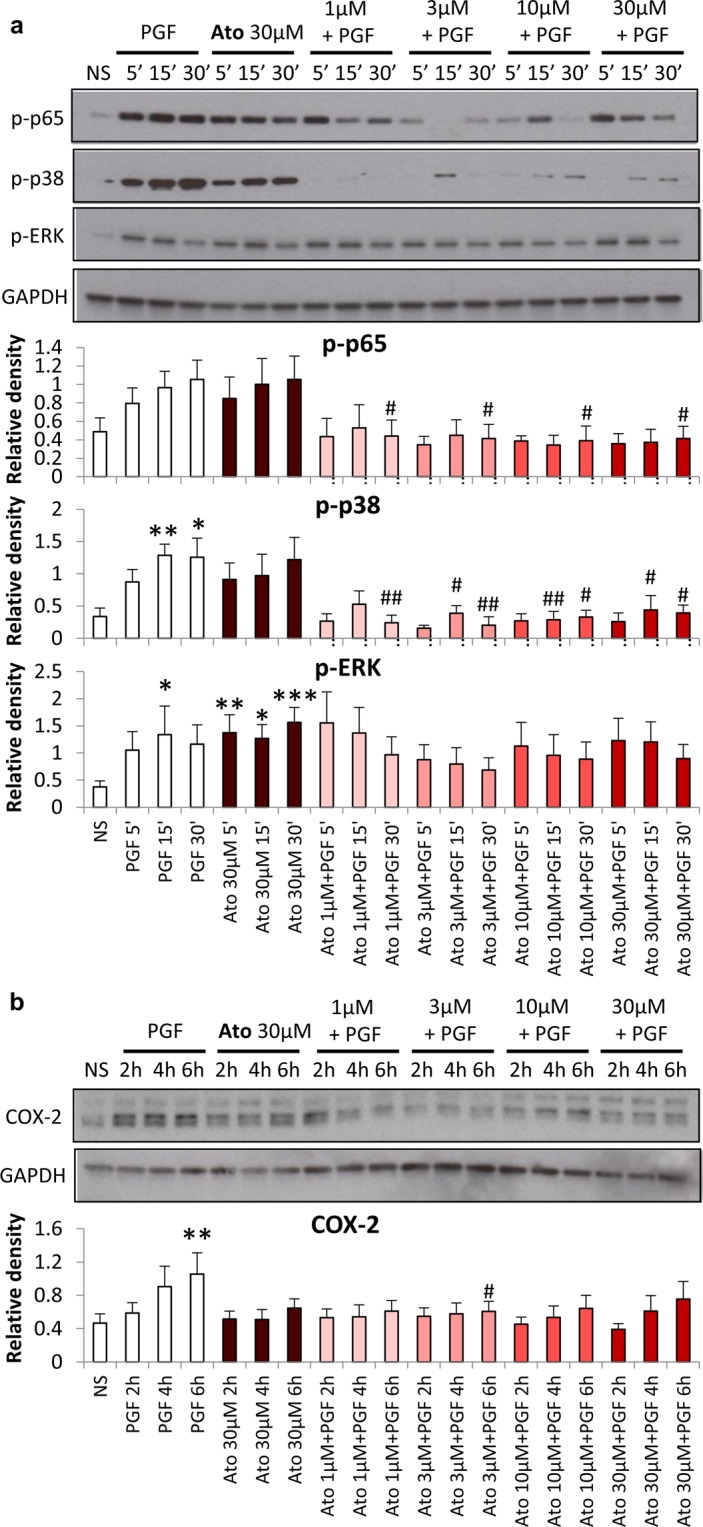
The effect of atosiban on PGF_2α_-induced pro-inflammatory response in myometrial cells. Pre-labour myometrial smooth muscle cells were treated with atosiban (Ato; 1, 3, 10, or 30 μM) and PGF_2α_ (1 µM) for 5 min, 15 min, 30 min, 2 h, 4 h and 6 h. Whole cell proteins were subjected to Western blot analyses with antibodies against phosphorylated NF-κB p65 subunit, ERK1/2 and p38 MAPK (**a**), as well as COX-2 (**b**), and matching densitometry analyses have been added below the representative blots. Membranes were probed with GAPDH to confirm equal loading (n = 6; *p < 0.05, **p < 0.01 compared with non-stimulated (NS); ^#^*p* < 0.05, ^##^*p* < 0.01 compared to PGF_2α_-treated, ANOVA).

Human myometrial smooth muscle cells were treated with PGF_2α_ in presence or absence of OTR-A, atosiban or nolasiban. As shown in previous studies, there was an increase in activation of p65 NF-κB subunit and MAPKs, p38 and ERK1/2, leading to upregulation of COX-2 with PGF_2α_ stimulation (Fig. 3a,b). Atosiban treatment alone was able to drive the activation of NF-κB and MAPKs, however, it resulted in significant inhibition of PGF_2α_-induced p-p65 (*p* < 0.05) and p-p38 MAPK (*p* < 0.01) expression (Fig. 3a). This inhibition translated downstream to suppress PGF_2α_-mediated COX-2 upregulation (*p* < 0.05) (Fig. 3b).

**Figure 4:**
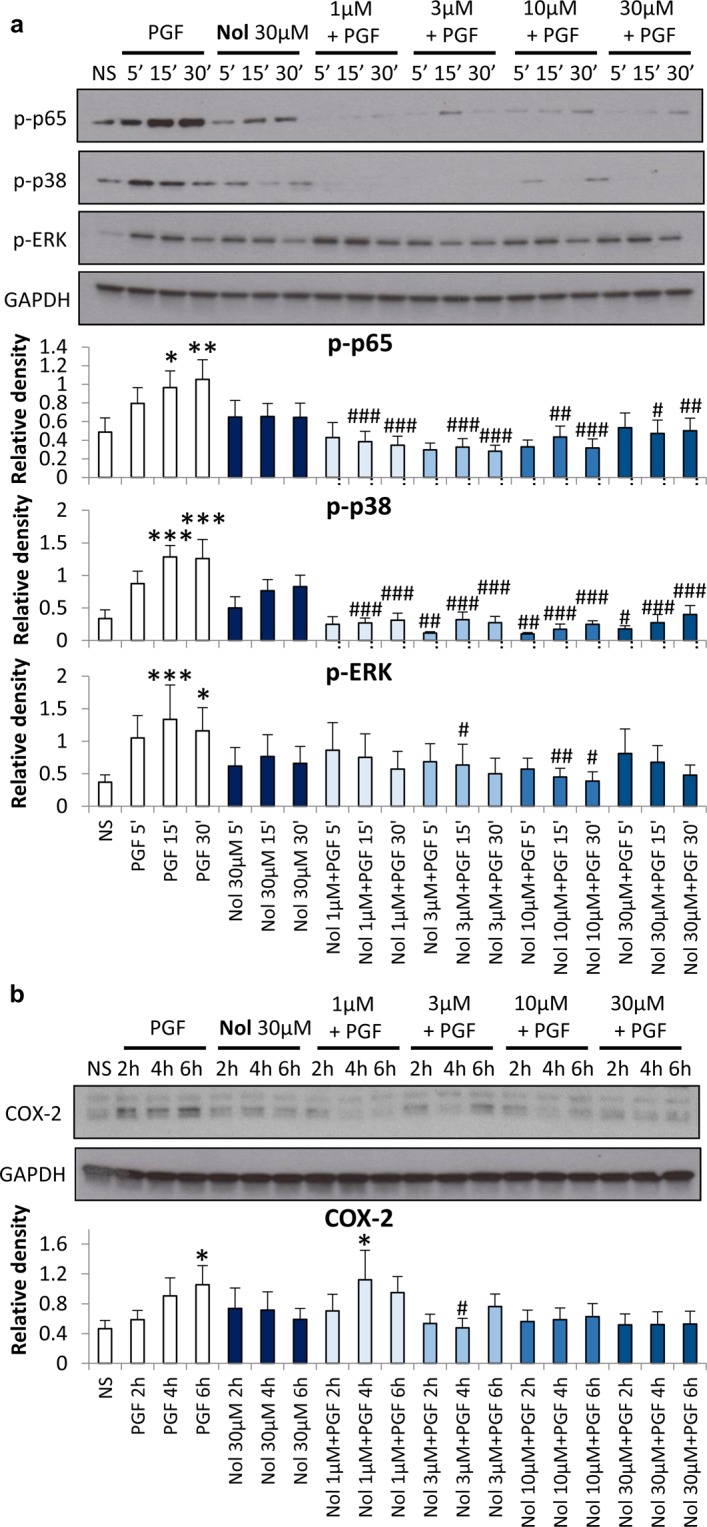
The effect of nolasiban on PGF_2α_-induced pro-inflammatory response in myometrial cells. Pre-labour myometrial smooth muscle cells were treated with nolasiban (Nol; 1, 3, 10, or 30 μM) and PGF_2α_ (1 µM) for 5 min, 15 min, 30 min, 2 h, 4 h and 6 h. Whole cell proteins were subjected to Western blot analyses with antibodies against phosphorylated NF-κB p65 subunit, ERK1/2 and p38 MAPK (**a**), as well as COX-2 (**b**), and matching densitometry analyses have been added below the representative blots. Membranes were probed with GAPDH to confirm equal loading (n = 6; *p < 0.05, **p < 0.01, ***p < 0.001 compared with NS; ^#^*p* < 0.05, ^##^*p* < 0.01, ^###^*p* < 0.001 compared to PGF_2α_-treated, ANOVA).

Similar to atosiban, nolasiban also led to significant inhibition of PGF_2α_-induced p-p65 (*p* < 0.001), p-p38 MAPK (*p* < 0.001), and p-ERK1/2 (*p* < 0.01) expression (Fig. 4a), as well as the downstream COX-2 expression (*p* < 0.05) (Fig. 4b). However, unlike atosiban, there was no increase in p-p65, p-p38 or p-ERK1/2 when treated alone.

## Discussion

We have shown that atosiban and nolasiban reduced not only OT-induced myometrial contractions in a dose-dependent manner but also PGF_2α_-induced myometrial contractions in an *ex vivo* myometrial contractility model. The two OTR-As have comparable potency in suppressing OT- and PG-augmented contractions. These effects appear to involve the suppression of agonist-driven increase in the intracellular calcium levels. The inhibitory effects of these OTR-As extended to affect PGF_2α_-induced activation of pro-inflammatory transcription factors, NF-κB and MAPKs, as well as the expression of contraction-associated-protein, COX-2.

Labour is defined clinically by the initiation of rhythmic uterine contractions. OT as well as PGs are potent stimulators of contractions and are commonly used to induce labour^23,24^. However, both OT and PGs are more than just a stimulator of myometrial contractions. They also play an important signalling role during the onset of labour, contributing to the transformation of the uterus by establishing a pro-inflammatory environment^25,26^. Therefore, the receptors of OT and PGs are attractive targets for development of drugs aimed at managing preterm labour.

Atosiban is a well-established OTR-A which is approved in Europe. Although the efficacy of atosiban in clinical trials is disputed^27^, it has been repeatedly shown to successfully inhibit OT-induced myometrial contractions *in vitro*^28–30^ by inhibiting inositol triphosphate (IP_3_) production with decrease in intracellular calcium mobilisation in the myometrial cell^12^ as we have shown in this study. Nolasiban is a non-peptide OTR-A, which has a higher selectivity for OTR than V1a receptors when compared to atosiban^14^. Here, we have demonstrated that the inhibitory effects of nolasiban on OT-augmented myometrial contractions and calcium response are similar to that of atosiban. However, unlike atosiban, nolasiban appears to have no biased agonistic effect on the pro-inflammatory signalling pathways^14^, thus highlighting the promising potential of nolasiban as a new tocolytic.

Our results demonstrate a robust concentration-dependent increase in myometrial contractions in presence of PGF_2α_ at 1 µM, albeit to a lesser extent than OT. This finding complements previous reports demonstrating significant increases in myometrial contractility in presence of PGF_2α_^30^. The levels of PGs have been found to increase in both amniotic fluid and the maternal blood during the onset labour^31^ and it is commonly believed that PGs directly stimulate myometrial contractions^32^. Thus PGF_2α_ agonists (eg. carboprost) are clinically used for the induction of labour as well as management of postpartum haemorrhage^33^. Based on the existing evidence, there is little doubt that PGs have an important role in the process of labour, however, some studies have reported PGs as ineffective contractile agents and implied an indirect role for PGs in inducing contractions^34,35^. It was suggested that PGs may exert their effects on contractility via a complex interplay of processes with various targets, possibly by inducing calcium influx, driving the production of other uterotonins and/or contraction-associated proteins, or by modulating the uterotonin receptivity of the myometrium^34,35^.

In binding assays, nolasiban at 10 µM showed no inhibition of FP receptors expressed in recombinant cell lines (data not shown, on file) indicating that nolasiban’s inhibitory effects on PGF_2α_-induced contractions and inflammatory responses are not mediated by direct inhibition of cognate FP receptors. This suggests that our results may be due to a possible crosstalk between OTR and FP receptor signalling as a result of receptor-receptor interaction and negative cooperativity, or overlap of downstream signalling pathways. The first evidence of interplay between OT and PG receptors was reported in 1980 when Baxi *et al*. found that low PGF_2α_ concentrations can enhance OT-induced uterine contractions^36^. A subsequent study suggested that this may be due to the increase in OTR binding affinity in term myometrium when stimulated with PGF_2α_^37^. More recently, further evidence of possible crosstalk between OT and PGF_2α_ at receptor level was provided. THG113.31, a non-competitive FP receptor antagonist peptide, led to a concentration-dependent suppression of OT-augmented contractions in term, non-labouring human myometrium^30^.

As tocolytics are designed to delay preterm labour, the main limitation of our study is the use of myometrial samples obtained from term pregnancies before labour and not from preterm labouring pregnancies. It is possible that the findings from this study may not translate directly to clinical settings, however, by limiting our sample collection to breech- or previous caesarean-indicated sections, we were able to study the *in vitro* effects of these OTR-As on myometrial contractions in a homogenous study population.

In conclusion, both OTR-As, atosiban and nolasiban, suppress OT- and PGF_2α_-induced calcium response to dose-dependently inhibit pregnant myometrial contractions. Moreover, the OTR-As suppressed the effect of PGF_2α_ on downstream signalling and gene expression indicating that there may be a shared mechanism between OTR and FP receptors. Further insight into this potential crosstalk/interaction between OTR and FP receptors would lead to identification of a novel approach to improve current tocolytic efficacy via combination tocolysis.

## Materials and Methods

### Human myometrial tissue collection

Myometrial tissues were collected from pregnant women undergoing scheduled elective caesarean section at term (38^+0^ - 40 weeks of pregnancy), prior to the onset of labour. All participating women were informed about the nature of the study in advance and informed written consents were provided with the approval from the Riverside Research Ethics Committee (RREC 3357). All experiments were performed in accordance with the committee’s guidelines and regulations. Women with multiple pregnancies or medical conditions such as diabetes, pre-eclampsia, obstetric cholestasis were not included in this study.

### Sample processing

The myometrial biopsies were obtained from the upper margin of the incision made at the lower segment of the uterus, and were stored in phosphate-buffered saline (PBS) at 4 °C for dissection. All samples were used for contractility experiments within 24 hours of collection. The biopsies were dissected into 8 longitudinal myometrial strips of 7 mm × 2 mm × 1 mm and mounted to thermostatically-controlled isolated organ baths (DMT Myograph 800MS) containing 7 ml of oxygenated (95% O_2_ and 5% CO_2_) Kreb’s solution (D-Glucose 2.0 g/L, Magnesium sulphate (anhydrous) 0.141 g/L, Potassium phosphate monobasic 0.16 g/L, Potassium chloride 0.35 g/L, Sodium chloride 6.9 g/L, Calcium chloride dihydrate 0.373 g/L, Sodium bicarbonate 2.1 g/L) at 37 °C, pH 7.4.

### Drugs and reagents

Dimethyl sulfoxide (DMSO) and atosiban were purchased from Sigma-Aldrich (Dorset, UK). PGE_2_ was from Tocris Bioscience (Bristol, UK), and PGF_2α_ from Cayman chemicals (Ann Arbor, MI, USA). OT (Syntocinon^®^) was from Alliance Pharmaceuticals (Chippenham, UK). Nolasiban was provided by ObsEva SA (Geneva, Switzerland).

Vehicle for OT was Kreb’s solution, atosiban was dissolved in double deionized water, and nolasiban in DMSO. The DMSO concentration was adjusted to 0.1% v/v in all dose formulations, and control wells for nolasiban experiments were treated to contain 0.1% v/v DMSO.

### Data acquisition

The longitudinal myometrial strips were subjected to 4 g (19.62mN) of tension to attain spontaneous contractions and the experiment was abandoned if more than 2 strips failed to initiate stable spontaneous contractions. After recording 20 min of stable basal contractions, OTR-A (atosiban or nolasiban; 6, 60 and 600 nM) or vehicle (equivalent volume) was added. The effect of OTR-A or vehicle was recorded for 10 min prior to cumulative dose responses for agonists (OT and PGF_2α_) that were added every 10 mins. OT concentrations ranged from 1 to 100 nM and PGF_2α_ concentrations ranged from 0.01 to 1 µM. Myometrial contractility was recorded by a force transducer with Powerlab and was analysed using LabChart5 with peak parameters extension (version 5.5.6; ADI instruments, Oxford, UK).

Acquired data were transferred from the datapad of the LabChart5 software for further analysis. The changes in the contractility in response to different treatments were measured by normalising to the basal spontaneous contractions of each strip and then to the equivalent time-point for the vehicle control strip.

### Measurement of intracellular calcium levels

Primary myometrial smooth muscle cells were isolated from non-labouring myometrial biopsies as previously described^14^. Once the cells reach confluence, they were passaged using 0.25% trypsin with 0.02% EDTA in PBS and seeded into 35 mm glass bottomed cell culture dishes (MatTek Corporation). Prior to treatment, cells were serum starved in 1% FCS DMEM overnight. For calcium mobilisation study, the cells were loaded with calcium sensitive Fluo-4 Direct buffer with 5 mM probenecid (Invitrogen) for 30 min at 37 °C in a 5% CO_2_ incubator and subsequently at room temperature for further 30 min. The dishes were then subjected for live cell imaging on the TCS-SP5 confocal microscope (Leica) with a x20 dry objective. Cells were imaged for 3 min and for 15 min after treatment with agonist/antagonist. The images were captured every 1.385 sec and the time-lapse movies were generated with the LAS-AF software (Leica). The changes in the fluorescence intensities were measured using open-source software, ImageJ/Fiji (US National Institutes of Health).

### Western blots

Non-labouring primary myometrial smooth muscle cells were treated with PGF_2α_ for 5 min, 15 min, 30 min, 2 h, 4 h or 6 h in presence/absence of atosiban or nolasiban (1, 3, 10 or 30 µM). Whole cell lysates were extracted using the modified radioimmunoprecipitation assay (RIPA) buffer^25^. Proteins were separated on a pre-cast SDS-polyacrylamide gel (Bio-Rad) and transferred to a PVDF membrane using the Trans-Blot® Turbo™ Blotting System following manufacturer’s protocol. All primary antibodies were optimized to minimize background and/or non-specific binding using full length blots (Supplementary Fig. S3). Once optimized, membranes were cut at 55 kDa and top halves were incubated in primary antibody, p-p65 (Cell Signalling) or COX-2 (Santa-Cruz Biotechnology), and bottom halves in p-p38 and p-ERK (Cell Signalling) overnight at 4 °C and in the appropriate HRP-tagged secondary antibody (Cell Signalling) for 1 h at room temperature the next day. Signal detection was done using ECL reagent (Bio-Rad) and ImageQuant LAS4000 (GE Healthcare). Equal loading was confirmed by blotting bottom halves of the membranes for GAPDH (Proteintech).

### Statistical analysis

For contractility and Western blot studies, all results were expressed as mean ± SEM with n = 6 experiments performed on myometrial samples from different patients. For measurements of intracellular calcium levels, all results were shown as maximum fluorescence ± SEM with n = 4. Two-way analysis of variance (ANOVA) and one-way ANOVA were conducted with *Bonferroni* and *Dunnett’s post-hoc* test, respectively, using Graphpad Prism (version 5.02; GraphPad Software, San Diego, CA, USA). Values were considered to be statistically significant at *p* < 0.05.

## Supplementary information

Supplementary Information

## Data Availability

All data generated or analysed during the currently study are available from the corresponding author on reasonable request.
