# Supplementary material for: Oxytocin Receptor Antagonists, Atosiban and Nolasiban, Inhibit Prostaglandin F2α-induced Contractions and Inflammatory Responses in Human Myometrium
Source: Sci Rep. 2019 Apr 8;9:5792. doi: 10.1038/s41598-019-42181-2 (PMC6453954; doi:10.1038/s41598-019-42181-2)
Supplement: Supplementary file 1 — Supplementary Information [file 41598_2019_42181_MOESM1_ESM.pdf]

Supplementary Information

Title: Oxytocin Receptor Antagonists, Atosiban and Nolasiban, Inhibit Prostaglandin $F_{2\alpha}$-induced Contractions and Inflammatory Responses in Human Myometrium

Authors: Sung Hye Kim, Lucia Riaposova, Hauwa Ahmed, Oliver Pohl, André Chollet, Jean-Pierre Gotteland, Aylin Hanyaloglu, Phillip R. Bennett and Vasso Terzidou*

***Corresponding author's contact information:** Dr. Vasso Terzidou, Tel: +44(0)20759 43720, Fax: +44(0)20 759 42148, Email: v.terzidou@imperial.ac.uk

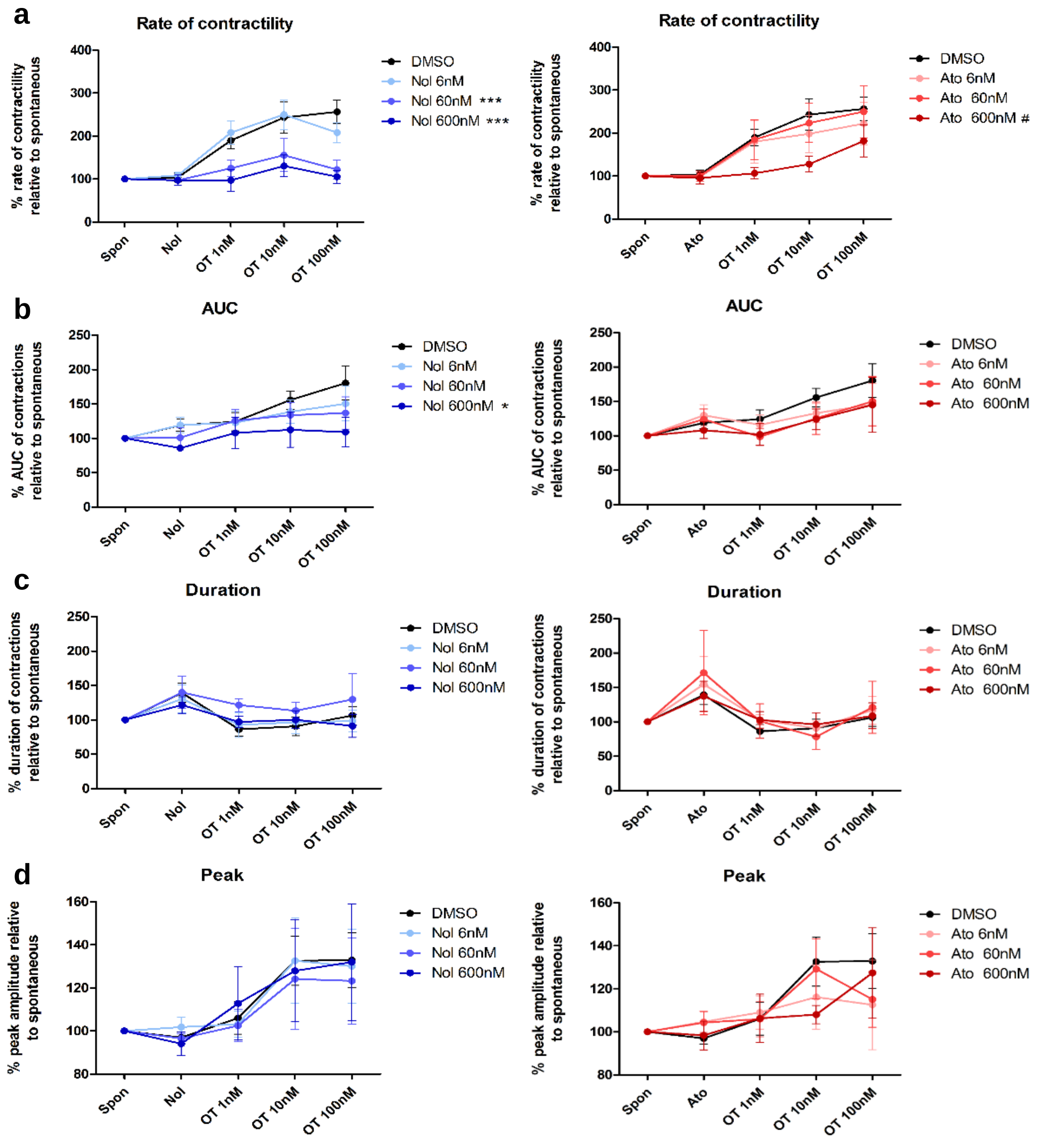

Supplementary Figure S1. The effect of atosiban and nolasiban on spontaneous and OT-induced myometrial contractions. Pre-labor lower segment myometrial biopsies were subjected to stretch force of 4g to attain spontaneous contractions. After 20 min of basal reading, vehicle control (DMSO), atosiban (Ato) or nolasiban (Nol) (6, 60, or 600nM) was added and its effect on spontaneous contractions was measured for 10 min. The effect of the atosiban or nolasiban upon OT was then measured by adding increasing concentrations of agonist (1, 10, and 100nM) at 10 minute intervals. For any individual strip, values for rate of contraction **(a)**, work per contraction (area under curve, AUC) **(b)**, contraction duration **(c)** and contraction peak **(d)** were measured for each experimental time point and re-expressed as a ratio to the baseline period measurements (n=6, * p<0.05, ***p<0.001 Nol vs DMSO; # p<0.05 Ato vs DMSO; ANOVA).

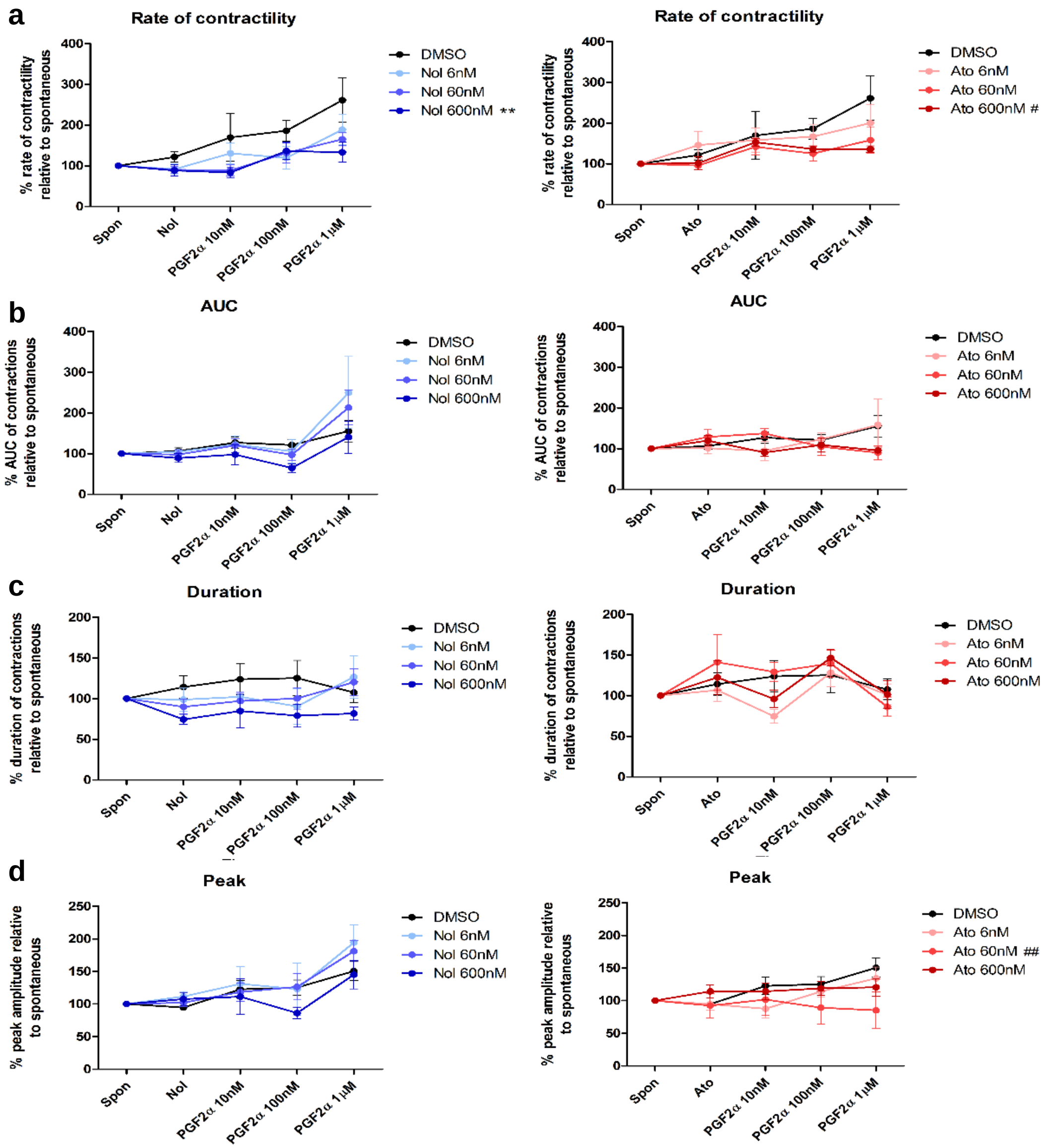

Supplementary Figure S2. The effect of atosiban and nolasiban on spontaneous and $PGF_{2\alpha}$-induced myometrial contractions. Pre-labor lower segment myometrial biopsies were subjected to stretch force of 4g to attain spontaneous contractions. After 20 min of basal reading, vehicle control (DMSO), atosiban (Ato) or nolasiban (Nol) (6, 60, or 600nM) was added and its effect on spontaneous contractions was measured for 10 min. The effect of the atosiban or nolasiban upon $PGF_{2\alpha}$ was then measured by adding increasing concentrations of agonist (10, 100, and 1000nM) at 10 minute intervals. For any individual strip, values for rate of contraction **(a)**, work per contraction (area under curve, AUC) **(b)**, contraction duration **(c)** and contraction peak **(d)** were measured for each experimental time point and re-expressed as a ratio to the baseline period measurements (n=6, ** p<0.01 Nol vs DMSO; # p<0.05, ## p<0.01 Ato vs DMSO; ANOVA).

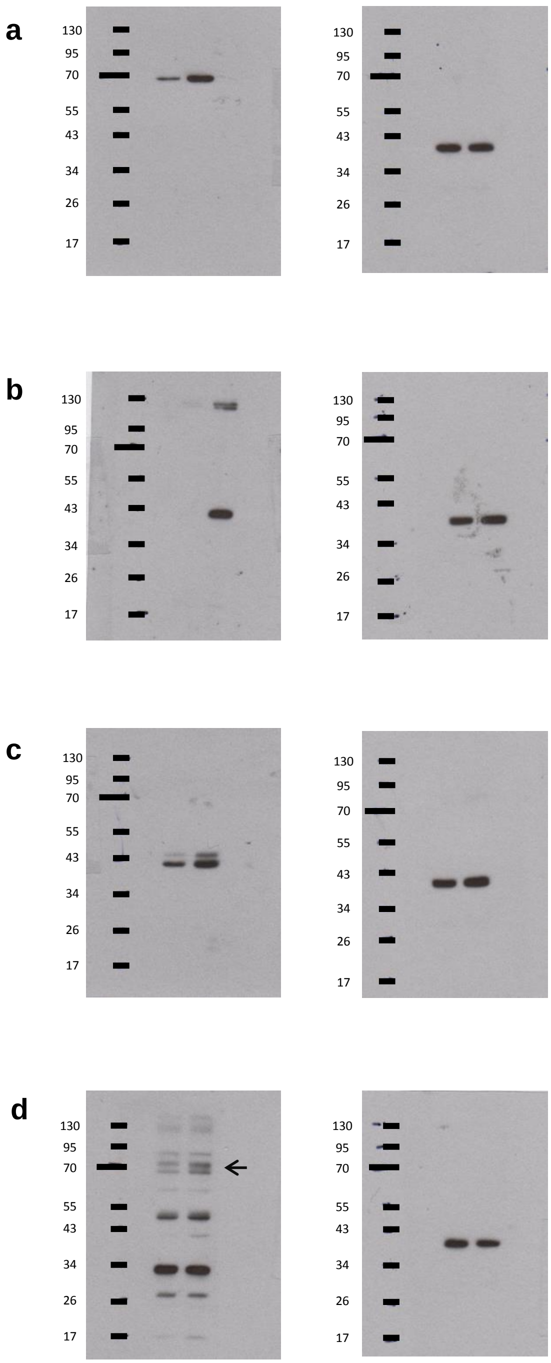

Supplementary Figure S3. Full length blots for all antibodies used in Western blot. Primary myometrial cells treated with PGF2α (1μM) for 30 minutes and 6 hours. Whole cell lysates were subjected to Western blot analyses for p-p65 **(a)**, p-p38 **(b)**, p-ERK1/2 **(c)** and COX-2 **(d)** using the full length blots to ensure that the antibody incubation steps have been optimized to minimize background and/or non-specific binding. Their corresponding GAPDH blots are shown on the right.

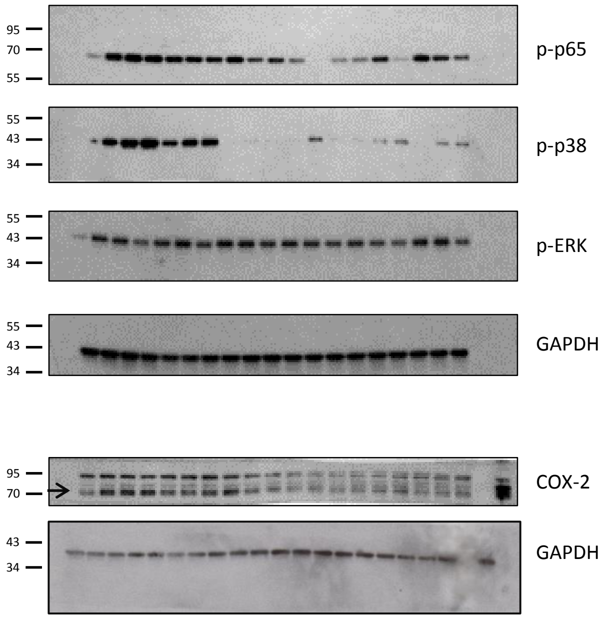

Supplementary information
Representative Blot Images for Figure 3.

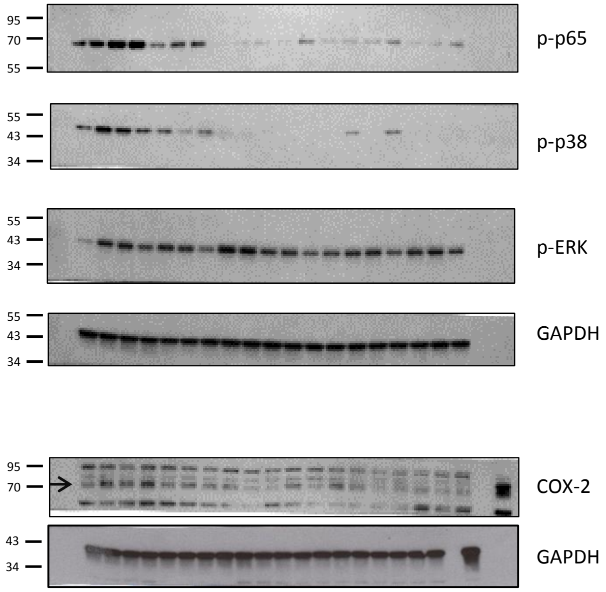

Supplementary information
Representative Blot Images for Figure 4.
